# 1-Cyano­methyl-1,4-diazo­niabicyclo­[2.2.2]octane tetra­chloridomanganate(II)

**DOI:** 10.1107/S160053681004047X

**Published:** 2010-10-20

**Authors:** Min Guo, Min Min Zhao

**Affiliations:** a Ordered Matter Science Research Center, College of Chemistry and Chemical Engineering, Southeast University, Nanjing 211189, People’s Republic of China

## Abstract

In the crystal structure of the title compound, (C_8_H_15_N_3_)[MnCl_4_], the Mn atom is coordinated by four chloride ligands in a slightly distorted tetra­hedral geometry. Each [MnCl_4_]^2−^ anion is connected to the 1-cyano­methyl-1,4-diazo­niabicyclo­[2.2.2]octane dications by N—H⋯Cl hydrogen bonds, forming chains parallel to [001].

## Related literature

For similar crystal structures of related compounds, see: Al-Far *et al.* (2008 ▶); Cai (2010 ▶). For the use of DABCO (1,4-diaza­bicyclo­[2.2.2]octa­ne) and its derivatives, see: Basaviah *et al.* (2003 ▶); Zhang, Cheng *et al.* (2009 ▶) and for its ferroelectric properties, see: Zhang, Ye *et al.* (2009 ▶); Ye *et al.* (2009 ▶).
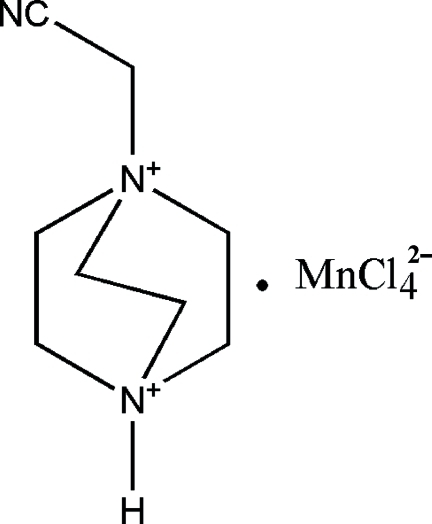


## Experimental

### 

#### Crystal data

(C_8_H_15_N_3_)[MnCl_4_]
*M* *_r_* = 349.97
Monoclinic, 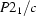

*a* = 8.373 (3) Å
*b* = 13.713 (6) Å
*c* = 12.188 (5) Å
β = 93.657 (8)°
*V* = 1396.6 (10) Å^3^
*Z* = 4
Mo *K*α radiation
μ = 1.69 mm^−1^
*T* = 298 K
0.2 × 0.2 × 0.2 mm

#### Data collection

Rigaku Mercury CCD diffractometer
Absorption correction: multi-scan (*CrystalClear*; Rigaku, 2005 ▶) *T*_min_ = 0.713, *T*_max_ = 0.721
14901 measured reflections
3181 independent reflections
2788 reflections with *I* > 2σ(*I*)
*R*_int_ = 0.035

#### Refinement

*R*[*F* ^2^ > 2σ(*F* ^2^)] = 0.033
*wR*(*F* ^2^) = 0.103
*S* = 1.11
3181 reflections
145 parameters
H-atom parameters constrained
Δρ_max_ = 0.66 e Å^−3^
Δρ_min_ = −0.52 e Å^−3^

### 

Data collection: *CrystalClear* (Rigaku, 2005 ▶); cell refinement: *CrystalClear*; data reduction: *CrystalClear*; program(s) used to solve structure: *SHELXS97* (Sheldrick, 2008 ▶); program(s) used to refine structure: *SHELXL97* (Sheldrick, 2008 ▶); molecular graphics: *SHELXTL/PC* (Sheldrick, 2008 ▶); software used to prepare material for publication: *SHELXL97*.

## Supplementary Material

Crystal structure: contains datablocks I, global. DOI: 10.1107/S160053681004047X/im2229sup1.cif

Structure factors: contains datablocks I. DOI: 10.1107/S160053681004047X/im2229Isup2.hkl

Additional supplementary materials:  crystallographic information; 3D view; checkCIF report

## Figures and Tables

**Table 1:** Hydrogen-bond geometry (Å, °)

*D*—H⋯*A*	*D*—H	H⋯*A*	*D*⋯*A*	*D*—H⋯*A*
N1—H1*C*⋯Cl2^i^	0.93	2.56	3.217 (2)	128
N1—H1*C*⋯Cl3^ii^	0.93	2.56	3.270 (2)	133

Symmetry codes: (i) 
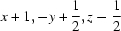
; (ii) 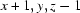.

**Fig. 1.**
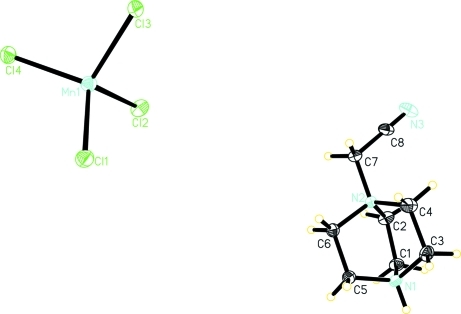
Molecular structure of the title compound, with the atomic numbering scheme. Displacement ellipsoids are drawn at the 30% probability level.

**Fig. 2.**
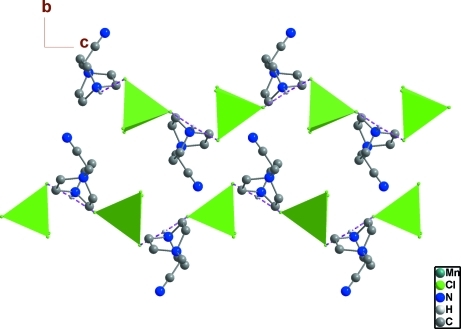
Structure of a layer of [ammonium-anion] chains cross-linked by hydrogen bonds. Dotted lines indicate hydrogen bonding. View is along the a axis.

## supplementary crystallographic information

### Comment

1,4-Diazabicyclo[2.2.2]octane (DABCO) is used as a effective organocatalyst for a large number of reactions because of its nucleophilicity (Basaviah *et al.*, 2003) and some of it's derivatives are ferroelectrics (Zhang *et al.*, 2009). This study is part of a systematic investigation of dielectric-ferroelectric materials (Ye *et al.*, 2009; Zhang *et al.*, 2009). The structural properties of related DABCO derivatives has been described earlier (Cai, 2010; Zhang *et al.*, 2009).

The asymmetric unit of the title compound is composed of cationic (C_8_H_15_N_3_)^2+^ and anionic (MnCl_4_)^2- ^ions (Fig 1). The Mn atoms are coordinated by four Cl atoms with very similar distances in the range of 2.366 (1) to 2.382 (1) Å. The Cl—Mn—Cl bond angles are between 101.58 (3) and 115.14 (3) ° which shows that the coordination polyhedron can be described as a slightly distorted tetrahedron. The ammonium groups of the organic cations are engaged in bifurcated hydrogen bonds to chlorine atoms of two (MnCl_4_)^2-^ anions. These weak N—H···Cl interactions cause the formation of a one-dimensional chain along the [0 0 1].

### Experimental

The ligand, 1-(cyanomethyl)-4-aza-1-azonia-bicyclo[2.2.2]octane bromide, was prepared as previously described (Cai, 2010).

MnCl_2_ × 4 H_2_O (0.001 mol, 0.197 g) and 2 ml 36% HCl were dissolved in MeOH (20 ml) and 1-(cyanomethyl)-4-aza-1-azonia-bicyclo[2.2.2]octane bromide (0.002 mol, 0.464 g) in the same solvent was added. The resulting solution was stirred until a clear solution was obtained. After slow evaporation of the solvent, colourless block crystals suitable for X-ray analysis were obtained in about 60% yield. The title compound has no dielectric disuniform from 80 K to 400 K, (m.p. > 401 K).

### Refinement

H atoms bound to carbon and nitrogen were placed in idealized positions [C—H = 0.97 Å and N—H = 0.93 Å] and allowed to ride on their parent atoms with (*U*_iso_(H) = 1.2 *U*_eq_(C,*N*).

### Crystal data

**Table:**

(C_8_H_15_N_3_)[MnCl_4_]	*F*(000) = 708
*M**_r_* = 349.97	*D*_x_ = 1.664 Mg m^−^^3^
Monoclinic, *P*2_1_/*c*	Mo *K*α radiation, λ = 0.71073 Å
Hall symbol: -P 2ybc	Cell parameters from 4022 reflections
*a* = 8.373 (3) Å	θ = 2.2–27.5°
*b* = 13.713 (6) Å	µ = 1.69 mm^−^^1^
*c* = 12.188 (5) Å	*T* = 298 K
β = 93.657 (8)°	PRISM, colourless
*V* = 1396.6 (10) Å^3^	0.2 × 0.2 × 0.2 mm
*Z* = 4	

### Data collection

**Table:**

Rigaku Mercury CCD diffractometer	3181 independent reflections
Radiation source: fine-focus sealed tube	2788 reflections with *I* > 2σ(*I*)
graphite	*R*_int_ = 0.035
Detector resolution: 28.5714 pixels mm^-1^	θ_max_ = 27.5°, θ_min_ = 2.2°
ω scans	*h* = −10→10
Absorption correction: multi-scan (*CrystalClear*; Rigaku, 2005)	*k* = −17→17
*T*_min_ = 0.713, *T*_max_ = 0.721	*l* = −15→15
14901 measured reflections	

### Refinement

**Table:**

Refinement on *F*^2^	Primary atom site location: structure-invariant direct methods
Least-squares matrix: full	Secondary atom site location: difference Fourier map
*R*[*F*^2^ > 2σ(*F*^2^)] = 0.033	Hydrogen site location: inferred from neighbouring sites
*wR*(*F*^2^) = 0.103	H-atom parameters constrained
*S* = 1.11	*w* = 1/[σ^2^(*F*_o_^2^) + (0.061*P*)^2^ + 0.074*P*] where *P* = (*F*_o_^2^ + 2*F*_c_^2^)/3
3181 reflections	(Δ/σ)_max_ = 0.001
145 parameters	Δρ_max_ = 0.66 e Å^−^^3^
0 restraints	Δρ_min_ = −0.52 e Å^−^^3^

### Special details

**Table:**

Geometry. All e.s.d.'s (except the e.s.d. in the dihedral angle between two l.s. planes) are estimated using the full covariance matrix. The cell e.s.d.'s are taken into account individually in the estimation of e.s.d.'s in distances, angles and torsion angles; correlations between e.s.d.'s in cell parameters are only used when they are defined by crystal symmetry. An approximate (isotropic) treatment of cell e.s.d.'s is used for estimating e.s.d.'s involving l.s. planes.
Refinement. Refinement of *F*^2^ against ALL reflections. The weighted *R*-factor *wR* and goodness of fit *S* are based on *F*^2^, conventional *R*-factors *R* are based on *F*, with *F* set to zero for negative *F*^2^. The threshold expression of *F*^2^ > σ(*F*^2^) is used only for calculating *R*-factors(gt) *etc*. and is not relevant to the choice of reflections for refinement. *R*-factors based on *F*^2^ are statistically about twice as large as those based on *F*, and *R*- factors based on ALL data will be even larger.

### Fractional atomic coordinates and isotropic or equivalent isotropic displacement parameters (Å^2^)

**Table:**

	*x*	*y*	*z*	*U*_iso_*/*U*_eq_	
Mn1	0.22628 (4)	0.23007 (3)	0.99138 (3)	0.02877 (13)	
Cl3	0.19440 (7)	0.39665 (4)	1.03991 (5)	0.03248 (16)	
Cl2	0.22006 (7)	0.23950 (5)	0.79599 (5)	0.03590 (17)	
Cl4	−0.00249 (7)	0.14312 (4)	1.04423 (5)	0.03416 (16)	
Cl1	0.47527 (7)	0.16000 (5)	1.05057 (5)	0.03580 (16)	
N1	0.8938 (2)	0.35643 (14)	0.19241 (15)	0.0270 (4)	
H1C	0.9919	0.3329	0.1718	0.032*	
N2	0.6277 (2)	0.42613 (13)	0.23649 (14)	0.0218 (4)	
C8	0.4225 (3)	0.55167 (18)	0.2010 (2)	0.0324 (5)	
C3	0.9196 (3)	0.45395 (19)	0.2452 (2)	0.0371 (6)	
H3A	0.9625	0.4991	0.1934	0.044*	
H3B	0.9956	0.4484	0.3083	0.044*	
C7	0.4681 (3)	0.46467 (17)	0.26521 (19)	0.0294 (5)	
H7A	0.3875	0.4146	0.2515	0.035*	
H7B	0.4721	0.4805	0.3429	0.035*	
C1	0.7902 (3)	0.36583 (19)	0.08854 (19)	0.0308 (5)	
H1A	0.7673	0.3018	0.0577	0.037*	
H1B	0.8451	0.4036	0.0352	0.037*	
C2	0.6363 (3)	0.4158 (2)	0.11339 (18)	0.0325 (5)	
H2A	0.5458	0.3780	0.0835	0.039*	
H2B	0.6314	0.4798	0.0793	0.039*	
C5	0.8155 (3)	0.28891 (18)	0.2681 (2)	0.0321 (5)	
H5A	0.8783	0.2851	0.3376	0.039*	
H5B	0.8088	0.2241	0.2363	0.039*	
N3	0.3839 (3)	0.61644 (16)	0.1482 (2)	0.0457 (6)	
C6	0.6493 (3)	0.32604 (19)	0.2868 (2)	0.0368 (6)	
H6A	0.5699	0.2817	0.2535	0.044*	
H6B	0.6347	0.3292	0.3650	0.044*	
C4	0.7606 (3)	0.4915 (2)	0.2813 (2)	0.0419 (6)	
H4A	0.7624	0.4928	0.3609	0.050*	
H4B	0.7427	0.5574	0.2545	0.050*	

### Atomic displacement parameters (Å^2^)

**Table:**

	*U*^11^	*U*^22^	*U*^33^	*U*^12^	*U*^13^	*U*^23^
Mn1	0.0269 (2)	0.0284 (2)	0.0308 (2)	0.00024 (14)	0.00125 (16)	−0.00150 (14)
Cl3	0.0358 (3)	0.0263 (3)	0.0360 (3)	−0.0027 (2)	0.0074 (3)	−0.0003 (2)
Cl2	0.0317 (3)	0.0471 (4)	0.0287 (3)	−0.0015 (3)	0.0010 (2)	−0.0043 (2)
Cl4	0.0312 (3)	0.0325 (3)	0.0390 (3)	−0.0035 (2)	0.0038 (3)	0.0021 (2)
Cl1	0.0310 (3)	0.0426 (3)	0.0339 (3)	0.0066 (2)	0.0024 (2)	0.0076 (3)
N1	0.0216 (9)	0.0309 (10)	0.0289 (10)	0.0021 (8)	0.0046 (8)	0.0022 (8)
N2	0.0219 (9)	0.0209 (9)	0.0227 (9)	−0.0001 (7)	0.0020 (7)	0.0008 (7)
C8	0.0296 (12)	0.0288 (12)	0.0384 (13)	0.0062 (10)	−0.0013 (10)	−0.0090 (11)
C3	0.0262 (12)	0.0370 (13)	0.0478 (15)	−0.0073 (10)	0.0017 (11)	−0.0057 (11)
C7	0.0247 (11)	0.0304 (11)	0.0337 (12)	0.0037 (9)	0.0068 (10)	−0.0017 (10)
C1	0.0312 (12)	0.0363 (13)	0.0252 (11)	0.0056 (10)	0.0037 (10)	0.0001 (9)
C2	0.0284 (12)	0.0468 (14)	0.0223 (11)	0.0065 (10)	0.0012 (9)	0.0013 (10)
C5	0.0283 (12)	0.0326 (12)	0.0361 (13)	0.0055 (10)	0.0066 (10)	0.0126 (10)
N3	0.0541 (15)	0.0317 (12)	0.0498 (14)	0.0123 (11)	−0.0081 (12)	−0.0093 (11)
C6	0.0364 (14)	0.0327 (12)	0.0430 (14)	0.0080 (10)	0.0148 (12)	0.0168 (11)
C4	0.0290 (13)	0.0350 (13)	0.0605 (17)	−0.0022 (11)	−0.0067 (12)	−0.0192 (13)

### Geometric parameters (Å, °)

**Table:**

Mn1—Cl1	2.3661 (10)	C3—H3B	0.9700
Mn1—Cl3	2.3789 (11)	C7—H7A	0.9700
Mn1—Cl4	2.3798 (10)	C7—H7B	0.9700
Mn1—Cl2	2.3822 (12)	C1—C2	1.507 (3)
N1—C5	1.488 (3)	C1—H1A	0.9700
N1—C3	1.494 (3)	C1—H1B	0.9700
N1—C1	1.494 (3)	C2—H2A	0.9700
N1—H1C	0.9325	C2—H2B	0.9700
N2—C7	1.500 (3)	C5—C6	1.512 (3)
N2—C4	1.504 (3)	C5—H5A	0.9700
N2—C6	1.509 (3)	C5—H5B	0.9700
N2—C2	1.513 (3)	C6—H6A	0.9700
C8—N3	1.132 (3)	C6—H6B	0.9700
C8—C7	1.464 (3)	C4—H4A	0.9700
C3—C4	1.518 (4)	C4—H4B	0.9700
C3—H3A	0.9700		
			
Cl1—Mn1—Cl3	115.14 (3)	N1—C1—C2	109.07 (18)
Cl1—Mn1—Cl4	115.01 (4)	N1—C1—H1A	109.9
Cl3—Mn1—Cl4	107.98 (3)	C2—C1—H1A	109.9
Cl1—Mn1—Cl2	106.83 (3)	N1—C1—H1B	109.9
Cl3—Mn1—Cl2	101.58 (3)	C2—C1—H1B	109.9
Cl4—Mn1—Cl2	109.34 (3)	H1A—C1—H1B	108.3
C5—N1—C3	110.32 (19)	C1—C2—N2	109.67 (18)
C5—N1—C1	108.90 (19)	C1—C2—H2A	109.7
C3—N1—C1	110.36 (19)	N2—C2—H2A	109.7
C5—N1—H1C	112.5	C1—C2—H2B	109.7
C3—N1—H1C	108.7	N2—C2—H2B	109.7
C1—N1—H1C	106.0	H2A—C2—H2B	108.2
C7—N2—C4	110.81 (18)	N1—C5—C6	109.26 (18)
C7—N2—C6	108.12 (17)	N1—C5—H5A	109.8
C4—N2—C6	109.14 (19)	C6—C5—H5A	109.8
C7—N2—C2	111.31 (17)	N1—C5—H5B	109.8
C4—N2—C2	109.57 (19)	C6—C5—H5B	109.8
C6—N2—C2	107.82 (18)	H5A—C5—H5B	108.3
N3—C8—C7	177.1 (3)	N2—C6—C5	109.40 (19)
N1—C3—C4	108.8 (2)	N2—C6—H6A	109.8
N1—C3—H3A	109.9	C5—C6—H6A	109.8
C4—C3—H3A	109.9	N2—C6—H6B	109.8
N1—C3—H3B	109.9	C5—C6—H6B	109.8
C4—C3—H3B	109.9	H6A—C6—H6B	108.2
H3A—C3—H3B	108.3	N2—C4—C3	109.6 (2)
C8—C7—N2	111.60 (19)	N2—C4—H4A	109.8
C8—C7—H7A	109.3	C3—C4—H4A	109.8
N2—C7—H7A	109.3	N2—C4—H4B	109.8
C8—C7—H7B	109.3	C3—C4—H4B	109.8
N2—C7—H7B	109.3	H4A—C4—H4B	108.2
H7A—C7—H7B	108.0		
			
C5—N1—C3—C4	−54.9 (3)	C3—N1—C5—C6	65.2 (3)
C1—N1—C3—C4	65.5 (3)	C1—N1—C5—C6	−56.1 (3)
C4—N2—C7—C8	72.5 (2)	C7—N2—C6—C5	−174.9 (2)
C6—N2—C7—C8	−167.9 (2)	C4—N2—C6—C5	−54.3 (3)
C2—N2—C7—C8	−49.7 (3)	C2—N2—C6—C5	64.6 (3)
C5—N1—C1—C2	66.0 (2)	N1—C5—C6—N2	−8.3 (3)
C3—N1—C1—C2	−55.2 (3)	C7—N2—C4—C3	−176.7 (2)
N1—C1—C2—N2	−8.3 (3)	C6—N2—C4—C3	64.4 (3)
C7—N2—C2—C1	−173.2 (2)	C2—N2—C4—C3	−53.5 (3)
C4—N2—C2—C1	63.9 (3)	N1—C3—C4—N2	−8.8 (3)
C6—N2—C2—C1	−54.8 (3)		

### Hydrogen-bond geometry (Å, °)

**Table:**

*D*—H···*A*	*D*—H	H···*A*	*D*···*A*	*D*—H···*A*
N1—H1C···Cl2^i^	0.93	2.56	3.217 (2)	128
N1—H1C···Cl3^ii^	0.93	2.56	3.270 (2)	133

Symmetry codes: (i) *x*+1, −*y*+1/2, *z*−1/2; (ii) *x*+1, *y*, *z*−1.
